# Community-based Participatory Research in the California Health Interview Survey

**Published:** 2005-09-15

**Authors:** E. Richard Brown, Sue Holtby, Elaine Zahnd, George B Abbott

**Affiliations:** University of California, Los Angeles, Center for Health Policy Research, UCLA School of Public Health; Public Health Institute, Santa Cruz, Calif; Public Health Institute, Oakland, Calif; California Public Health Association North, Sacramento, Calif

## Abstract

**Introduction:**

The California Health Interview Survey, the largest state health survey in the United States, uses community-based participatory research principles to develop each cycle. Other large-scale health surveys rarely include participatory research approaches. Every 2 years, the California Health Interview Survey generates state and local population-based data on health insurance coverage, access to health care, chronic disease prevalence and management, health behaviors and disease prevention, and other health issues in California. The survey is used for policy and program development, advocacy, and research.

**Methods:**

The development of the California Health Interview Survey involves more than 145 people from more than 60 state and local policymaking bodies, public health agencies, advocacy groups, research organizations, and health care organizations. They participate as volunteers in an advisory board, on technical advisory committees, and in work groups that interact with California Health Interview Survey research staff in an accountable advisory process that shapes survey topics, measures, and sample design and determines languages selected for translation. Survey results and data are provided to the communities involved in the survey.

**Results:**

California Health Interview Survey data have been widely used by local, state, and national public health leaders, policymakers, advocates, and researchers to improve access to health insurance and health care services and to develop and target prevention programs for obesity and chronic illnesses.

**Conclusion:**

The California Health Interview Survey participatory research model has been an effective approach to planning and implementing a health survey and should be considered by developers of other large health surveys.

## Introduction

The use of participatory research has been recognized as an effective way to increase the accountability of researchers to the communities they study. Health-related participatory research is designed to enhance research relevance and quality and empower communities to use the results to improve the conditions that affect their health (1-3). Often called *community-based participatory research *(*CBPR*) or *community-based research,* participatory research is a "collaborative approach to research that equitably involves . . . community members, organizational representatives, and researchers in all aspects of the research process" (2). Proponents of CBPR methods emphasize the role of community members as agents of change and consider participatory research to be an effective way to encourage and initiate community change by the community members who are affected. Participatory research has been shaped by numerous influences, including the action research of Kurt Lewin and the Latin American liberation movements influenced by Paulo Freire (4).

Some of the key elements that characterize CBPR include recognizing a community (a geographic community or a community of identity) as an essential research partner; building collaborative partnerships in all phases of the research (from problem definition to data collection to dissemination of results); gathering information that can be used to take steps to improve health; disseminating findings and knowledge gained from the research to all partners involved; and contributing to the ability of community members to work together to improve health (2). Participatory research methods have been used primarily in local community studies. The smaller scale of community studies enables researchers and community leaders to collaborate in person while planning and conducting studies, analyzing data, presenting findings, and developing publications.

However, large-scale health surveys rarely include participatory research methods. Community organizations and leaders are seldom involved in planning and developing sample design and content for population-based surveys sponsored by government agencies or private organizations. Large government agencies conduct surveys to generate data to be used to assess policies and develop new ones; they also are typically more accustomed to collaborating with other government agencies and using a top-down decision-making style than they are to working with communities to determine who and what is surveyed. Private organizations usually sponsor these large surveys to develop population-based information to meet specific research goals rather than to provide data for community organizations and local agencies. A recent comprehensive review of CBPR studies found no examples of large-scale health surveys that involved CBPR methods (5; E. Eng, oral communication, March 2005).

The California Health Interview Survey (CHIS), the nation's largest state health survey, incorporated a modified model of CBPR. The survey's use of CBPR far exceeds participatory research used in previous large-scale surveys. The CHIS is a biennial population-based health survey. It involved more than 56,000 households in 2001 and more than 42,000 households in 2003. The CHIS collects information on health insurance coverage, access to health care, chronic disease prevalence and management, health behaviors and disease prevention, and other health issues. The survey is a collaborative effort of the University of California, Los Angeles (UCLA) Center for Health Policy Research (referred to as *the Center* in this article), the California Department of Health Services (DHS), and the nonprofit Public Health Institute (PHI). These organizations created a structure and process that involve a broad range of constituencies that participate in the planning of each survey cycle and are provided with data and analysis assistance to independently interpret and apply survey findings. The CBPR elements of the CHIS are key components of its mission to be a valuable public service accountable to various communities and responsive to their needs. (For more information, go to http://www.chis.ucla.edu.)

In this article, we describe a participatory model for use in large health surveys, beginning with the limited-participation planning project that led to the CHIS, the substantial participation that guides the planning of each CHIS cycle, and the extensive dissemination of data back to participating communities and constituencies.

## Methods

### Development of the California Health Interview Survey

The first CHIS (CHIS 2001) was the product of a 3-year planning project that included a technical assessment component and several outreach activities that are consistent with the limited level of constituency participation found in many public health needs assessments (6). The planning project incorporated input from many state and local public health agencies, health care organizations, the academic community, and advocacy groups through a series of public meetings, key informant interviews, and questionnaires sent to potential data users. Project staff members from the PHI, the Center, and the DHS used the outreach results to develop the CHIS (UCLA Center for Health Policy Research, unpublished data, December 1997).

The CHIS sample design reflects the developers' responses to participant suggestions. Many public health and advocacy respondents expressed a need for data on California's ethnically diverse population, with a specific focus on smaller ethnic and racial groups (such as Asian ethnic groups and American Indians/Alaska Natives) that are seldom adequately represented in national or state population-based health surveys. At the same time, given the size of California's population and the physical size of the state itself, local health departments and state and community advocacy groups emphasized their need for local population data capable of reflecting estimates for small geographic areas. In response to both of these requests, the CHIS sample was designed to yield estimates for most counties in California and for the state's major ethnic and racial groups, as well as for numerous smaller racial and ethnic populations. The sample design evolved into a multistage random-digit–dial telephone survey with 41 geographically defined sampling strata, a large sample, and oversamples of several ethnic populations.

Feedback on the content also influenced the final design. Respondents suggested including many health topics that are not usually addressed in other California population-based surveys or for which statewide data but no local data exist; such topics include gun violence, sexual orientation, and access to health care. This feedback confirmed preliminary plans for developing an omnibus public health survey that covered a broad range of health topics, with an ongoing core set of topics and other special topics that rotated in and out of the survey over successive cycles.

Respondent feedback also influenced how often the survey would be conducted. Potential data users reported that it would be sufficient to collect data about key indicators every 2 years rather than every year. Therefore, the survey was designed as a biennial survey, with more resources being used to recruit the large sample size needed to achieve the estimation goals rather than being used for annual data collection, which would have been far more expensive than a biennial survey.

Thus, the planning project followed a widely used needs assessment model. Community participants focused on providing input that was structured, analyzed, and used by the project staff. Although respondents who provided input during the planning process were not involved in decision making, their feedback influenced the survey's content, sample design, and sample size; the populations and geographic areas sampled; and the frequency of data collection.

### The California Health Interview Survey participatory model

At the conclusion of the planning project, a new and more extensive ongoing participatory structure and process were created to develop policy and content for the new survey, a participatory model shown in the Figure. The three planning organizations established a formal collaboration, with the Center as the lead organization and DHS and PHI serving as partners. The organizations also agreed to develop a structure that involved advocacy groups, state and community health care organizations, and public agencies in planning all aspects of each CHIS cycle.

**Figure:**
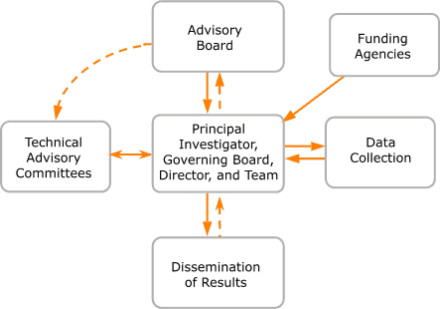
The California Health Interview Survey participatory model.

The collaborating organizations established a governing board — the ultimate decision-making body — in which the lead CHIS person from each organization was represented. The CHIS principal investigator leads the *CHIS team,* which includes the CHIS director, senior CHIS staff members at the Center, and staff members from PHI and DHS. The CHIS team is responsible for designing and managing the survey, collecting data, disseminating data and results, and raising the more than $12 million required to implement each 2-year CHIS cycle. The CHIS team incorporates the input of a wide array of stakeholders to guide the development of each survey.

### Participatory planning

#### The advisory board

The governing board created the CHIS advisory board (Figure) to provide ongoing policy guidance for all phases of the survey. The advisory board has more than 25 members, and its first chairperson was the director of DHS. Its current chairperson is the governor of California's cabinet-level secretary for health and human services. The advisory board members include directors of three state health agencies and chief executive officers or presidents of statewide associations of local health departments, community clinics, rural health care providers, hospitals, and health plans; public health officers from two large California counties; members of the research arm of the legislature; members of 12 advocacy organizations representing populations of color, low-income populations, children, and people with disabilities; members of a foundation; and representatives from the University of California health division.

The advisory board meets quarterly and recommends issues to address, topics to include in the survey, sampling goals, dissemination goals, and funding strategies. Although the advisory board can only recommend policy to the governing board, if the governing board chooses not implement a particular recommendation, it explains the reason to the advisory board. Although such a relationship could lead to tension over the authority and role of the advisory board, the responsiveness of the CHIS team to the advisory board has prevented any such conflict.

#### Technical advisory committees

Formal technical advisory committees (TACs) were created to advise the CHIS team on specific content and measurement issues (Figure). Individual TACs are responsible for providing advice on the adult, adolescent, and child questionnaires; sample design and survey methodology; and multicultural issues. For each CHIS cycle, the multicultural issues TAC provides advice on which specific ethnic groups need formal language translations, which groups need culturally specific interview adaptations, and measurement issues related to ethnicity, acculturation, and discrimination. Additional working groups are formed as needed to focus on more specific content, measurement, and population issues, including groups on women's health, diet and nutrition, physical activity, disability, and aging.

The questionnaire topics for the survey are proposed and discussed at each relevant TAC meeting. Before the meetings, the CHIS team distributes a list of topics that were included in previous surveys and a list of topics that had been recommended and discussed but not included. Each TAC member can suggest new topics, recommend including or not including previously used topics, suggest new or different question formats, and discuss all topics and specific questions being considered. By the conclusion of the meeting, the TAC has discussed all survey topics and ranked them in order of importance according to how well they support policy advocacy and development or research needs for the relevant population. Based on the rankings of the TAC, the topics are then developed by the CHIS team for inclusion in the survey, but the amount of interview time available ultimately affects the number of topics included. TAC members are encouraged to access a Web site to review and comment on in-progress drafts.

Although the TACs handle technical issues, their members have a broad range of expertise and include staff members of advocacy, public health, and health care delivery organizations, as well as researchers affiliated with major universities and research organizations. The TACs meet once or twice during the planning process. The CHIS encourages in-person participation by paying the transportation costs of those who must travel to attend the meetings. Like the advisory board, TACs serve as advisors to the CHIS team. Few barriers to recruiting and retaining TAC members have emerged, largely because the members know they have an impact on the survey. Their input is reflected in the survey questionnaires, sample design, and other elements of each survey.

During the development of the CHIS 2001, more than 100 individuals from 54 separate scientific, professional, advocacy, and community-based organizations participated in the advisory board and five TACs, and another 20 people participated in the more narrowly focused work groups. For the CHIS 2003, more than 145 individuals from more than 60 organizations participated.

#### Funders

Major funders for the survey play a key role in its development (Figure). Because of the survey's high cost, its survival depends on the support of government agencies and foundations. Although California's commitment to the survey is strong, the state only funds a quarter of the survey's costs. The CHIS 2001 received more than $1 million from each of five funders, and the CHIS 2003 received more than $1 million from each of four funders. In addition, five other funders provided substantially less than $1 million each in 2001, as did six funders in 2003.

The CHIS's dependence on multiple funders clearly affects the survey team's interactions with the CHIS advisory board and the TACs. The need for multiple funders could result in substantial, potentially conflicting, influences. However, because DHS is a CHIS partner and its core funder, its commitment to supporting the participatory process and involving its own program and research staff members in that process provides latitude for the CHIS team to be responsive to other funders and the broader constituency. The CHIS team also seeks funding to support the topic areas and population groups identified as priorities by the CHIS advisory board and the TACs. In addition, all funders are required to contribute to overall content and sample objectives.

#### Reconciling interests: examples of the California Health Interview Survey process

Despite the potential for conflict between the advisory component of CHIS (the advisory board and TACs) and the CHIS team and funders, their goals have been remarkably similar. For example, advocates and researchers on the advisory board and the multicultural issues TAC strongly recommended oversampling Asian ethnic groups and American Indians/Alaska Natives — recommendations that were unanimously supported by the advisory board and TACs. Two major funders were enthusiastic about supporting these decisions: a federal agency with research interest in the populations and a foundation that was urged by advocates to support data collection on Asian ethnic groups. The CHIS team also secured additional funding from the U.S. Indian Health Service for oversampling of American Indians/Alaska Natives.

The importance of being represented during and participating in the survey's development is illustrated by the choice of groups that were oversampled. Asian ethnic groups and American Indians/Alaska Natives are the only groups that have been oversampled. Latino and African American advisory group members did not advocate oversampling of the populations because sufficient sample sizes were expected to be generated. No advisors suggested oversampling predominantly white ethnic subgroups such as Armenians or Russians, even though a significant percentage of their populations are immigrants and are likely to have significant health issues. The issue was discussed at the multicultural issues TAC meetings. However, because no advocacy groups from these populations were represented in any of the advisory groups, members of other populations argued for other oversampling priorities. Thus, those who were more involved were able to understand the survey's resource limitations and were satisfied with the anticipated outcomes, whereas the population groups that were not well-represented had no real opportunity to express their views, a common limitation in community-based participatory research (4).

Explicit, formula-based criteria were used to develop various facets of the survey. For example, the CHIS team worked with the multicultural issues TAC to develop quantitative criteria for selecting the non-English languages into which the survey would be translated. Languages for translation were selected based on the number of people from the language-related ethnic group that was predicted to be in the CHIS sample and the percentage of the group that was "linguistically isolated" (i.e., that lived in a household in which no member older than age 14 years could speak English well) (7). The final selection of languages was based on these criteria and was acceptable to the advisory groups and funders alike.

The sample and questionnaires for each survey cycle are developed by the CHIS team but guided by recommendations of several key advisory bodies that represent a broad range of user constituencies from state and local governments, public health organizations, health care providers, and advocacy groups. Ensuring that all relevant participants know how decisions are being made combined with explaining resource constraints and variations from advisory body recommendations result in an atmosphere of trust between the CHIS team and the advisory groups.

## Results

### Data dissemination

Constituencies that participate in the CHIS ultimately benefit from its results. Consistent with the CHIS public service mission and its participatory research model, substantial resources are used to make CHIS results available and accessible to a wide range of constituencies (Figure). Historically, data analysis results have been available and useful only to people and organizations with significant technical abilities. Community and advocacy groups and many local health departments face many obstacles when they try to use health data for policy and development work. Barriers include limited availability of relevant data, an inability to analyze available data, and limited knowledge of how to use the data effectively.

The Center has developed multiple programs to democratize access to its data and to the analytic tools that transform the data into useable information. CHIS 2001 and 2003 data have been disseminated to state and county health departments, policymakers, health researchers, community-based organizations, advocacy groups, and the public through publications, fact sheets, data files, local workshops, and an easy-to-use online data query system.

### Publications using California Health Interview Survey data

Publications written for and disseminated to broad audiences offer easy access to CHIS results that can be adapted to policy development, advocacy, and funding proposals. The Center has published nine major policy research reports based on CHIS 2001 data, as well as several four- to six-page policy briefs and two-page fact sheets. Each publication addresses issues of disparities by race and ethnicity, social position, residence (urban or rural), and (if sample size permits) geographic differences within the state.

Publications using CHIS 2001 data are being widely used to develop state and local health policy, particularly to expand health insurance programs, asthma education programs, and food stamp access. A Listserv message and usually a press release are used to announce the release of a new publication. Although the publications are also distributed in print form, the Internet is the primary method of dissemination. For example, within 20 months of its release, a particular diabetes report was downloaded from the Center's Web site more than 31,000 times, and within 21 months of its release, a policy brief on food insecurity and hunger in California was downloaded more than 50,000 times. A report with estimates for a wide range of health indicators for adults, adolescents, and children was downloaded more than 32,000 times in its first 6 months.

### *Ask*CHIS: open access to data and analysis

One of the most innovative CHIS tools is a uniquely user-friendly online query system called *Ask*CHIS, which can be used by anyone with Internet access. Users can request data about various health topics and obtain detailed descriptive statistics based on CHIS data tailored to their needs. For example, a user could request information on the age at which African Americans living in Orange County in 2001 were first diagnosed with diabetes. The results are provided in bar graphs, pie charts, and downloadable Excel spreadsheets and include confidence intervals that take into account the survey's complex design. *Ask*CHIS makes the data much more useful for community-based organizations, policymakers, and public health officials because it offers highly customized survey results. As illustrated by the example, the system provides detailed geographic and demographic data while protecting the confidentiality of respondents through a statistical algorithm that suppresses estimates that could inadvertently lead to identification of individual respondents. The user is able to e-mail the results to colleagues and policymakers.

One of the goals of *Ask*CHIS was to maximize usability for basic community-level users but still provide value to advanced users. Comments volunteered to CHIS staff via a "feedback" button and a recently completed user survey have been enthusiastic, even when offering suggestions for improvements. To date, more than 6000 *Ask*CHIS users have registered, with an average of 16 queries per user; according to one user, it is "an awesome resource," and another describes it as "the trusted gold standard in policy circles" (*Ask*CHIS survey, unpublished data, 2004).

*Ask*CHIS is an example of a tool that can assist less technically sophisticated community-based advocates, policymakers, and organizations that have limited analytic resources by allowing universal access to data and analytic methods that previously have been the exclusive domain of researchers. This type of data sharing with participating communities is a hallmark of participatory research.

### Public-use files and services for researchers

Electronic public-use data files, including supporting documentation, are available for free download from the CHIS Web site. More than 550 people have completed electronic confidentiality agreements and downloaded data files within 18 months from the time they became available. Although the public-use files are useful only to researchers and health policy analysts with statistical analytic skills and equipment, numerous organizations in California that serve relevant populations either have or collaborate with researchers and data analysts. The data files are therefore an additional resource and benefit for participating groups and the populations they represent. Researchers' and analysts' growing familiarity with the CHIS has enhanced the perception among funding agencies that the CHIS is important and worth funding.

### Local workshops

To introduce members of community-based organizations and agencies to CHIS publications and *Ask*CHIS, the CHIS organizes workshops in many areas throughout the state. About two thirds of the workshops available are for nontechnical people in local organizations and agencies that have limited or no analytic resources. About one third of workshops, which include introductions to CHIS public-use data files, are intended for researchers and analysts in local agencies, organizations, and academic organizations.

## Discussion

Promoting access to and use of CHIS publications, *Ask*CHIS, and data files allows data and results to be accessible to the state and local organizations that planned and developed each survey. The wide use of CHIS dissemination tools demonstrates the relevance and importance of the survey to a broad range of constituencies. CHIS results are being used at state and local levels to shape and increase public health insurance coverage, track asthma rates and develop asthma-control policies and programs, encourage diabetes prevention by reducing obesity and focusing on diabetes management, and develop policies and programs to support children's and adolescents' health and development. Results are also used to identify and track disparities in health and access to care based on income, geographic location, racial and ethnic groups, and other social characteristics. In addition, CHIS data are being used in epidemiologic research to increase understanding of individual and environmental factors that influence asthma and other health conditions and access to health services. The many different methods of disseminating CHIS data and results ensures that groups with varying technical abilities can benefit from the survey.

The CHIS model of CBPR optimizes participation in the development and implementation of large-scale health surveys, which have traditionally involved planning processes that reflect only the views of the agencies and researchers directly involved in sponsoring and conducting the survey. This participatory research model is a hybrid approach that other large health surveys can use. The model ensures that 1) the survey is relevant to the communities that plan it, 2) the survey appropriately measures factors related to community needs, and 3) data and results are available and accessible to the relevant communities and their advocates. The community and advocacy participation in CHIS planning and development is not as extensive as the participation in local community-based studies that use the CBPR approach. However, the CHIS model is a viable CBPR approach, incorporating the flexibility suggested by Israel et al (2) and the collaborative approach summarized by Viswanathan et al (5).

The advisory roles of advocacy, service, and policy organizations in the planning and design of the CHIS allows them to shape each survey, whereas the CHIS research team obtains funding and manages the questionnaire development and data collection. The dissemination of results directly benefits groups that participate in the planning process and indirectly benefits the communities and populations that participate as respondents. The use of the data for policy development and advocacy makes the CHIS a valuable tool for public health professionals, and it deserves continuing support.
